# Announcement

**Published:** 1898-01

**Authors:** 


### ANNOUNCEMENT.

It gives us pleasure to announce to our readers that Dr. Charles Dunbar Roy has purchased an interest in this JOURNAL, and that he will be associated with its editorial management from this issue.

Dr. Roy is already well and favorably known from a number of important contributions to medical literature, several of which have appeared in these pages in the past. We think the JOURNAL is fortunate in securing the services and influence of Dr. Roy. The division of editorial work which follows this arrangement will be highly advantageous to the JOURNAL. L. B. G.

---

WE desire during the present year to make THE JOURNAL more readable and entertaining to its subscribers than ever before. We are going to make its pages contain original matter of the very first quality, and especially to consist of articles which will interest and benefit the general practitioner. We desire to have more local and State news items. We invite all our readers to send us queries, articles, news items from any portion of the State or country, so that there will be things of interest for every physician.

